# Analysis of ADORA2A rs5760423 and CYP1A2 rs762551 Genetic Variants in Patients with Alzheimer’s Disease

**DOI:** 10.3390/ijms232214400

**Published:** 2022-11-19

**Authors:** Vasileios Siokas, Dimitra S. Mouliou, Ioannis Liampas, Athina-Maria Aloizou, Vasiliki Folia, Elli Zoupa, Anastasios Papadimitriou, Eleftherios Lavdas, Dimitrios P. Bogdanos, Efthimios Dardiotis

**Affiliations:** 1 Laboratory of Neurogenetics, Department of Neurology, University Hospital of Larissa, Faculty of Medicine, School of Health Sciences, University of Thessaly, 41100 Larissa, Greece; 2 Lab of Cognitive Neuroscience, School of Psychology, Aristotle University of Thessaloniki, 54124 Thessaloniki, Greece; 3 Larisa Day Care Center of People with Alzheimer’s Disease, Association for Regional Development and Mental Health (EPAPSY), 15124 Marousi, Greece; 4 General Hospital of Lamia, 35100 Lamia, Greece; 5 Department of Biomedical Sciences, University of West Attica, 12243 Athens, Greece; 6 Department of Medical Imaging, Animus Kyanoys Larisas Hospital, 41222 Larissa, Greece; 7 Department of Rheumatology and Clinical Immunology, University Hospital of Larissa, Faculty of Medicine, School of Health Sciences, University of Thessaly, 41100 Larissa, Greece

**Keywords:** Alzheimer’s disease, caffeine metabolism, oxidative stress, polymorphism, genetics, ADORA2A rs5760423, CYP1A2 rs762551

## Abstract

Various studies have been conducted, exploring the genetic susceptibility of Alzheimer’s disease (AD). Adenosine receptor subtype A2a (ADORA2A) and cytochrome P450 1A2 (CYP1A2) are implicated in pathways such as oxidative stress and caffeine metabolism, which are associated with AD. The aim of this study was to explore for any potential association between the ADORA2A rs5760423 and the CYP1A2 rs762551 genetic variants and AD. A case–control study was performed with a total of 654 subjects (327 healthy controls and 327 patients with AD). Five genetic models were assumed. We also examined the allele–allele combination of both variants. The value of 0.05 was considered as the statistical significance threshold. A statistically significant association was found between ADORA2A rs5760423 and AD, as the “T” allele was associated with increased AD risk in recessive (OR = 1.51 (1.03–2.21)) and log-additive (OR = 1.30 (1.04–1.62)) genetic modes. In the codominant model, the TT genotype was more prevalent compared to the GG genotype (OR = 1.71 (1.09–2.66)). The statistical significance was maintained after adjustment for sex. No association between CYP1A2 rs762551 or allele–allele combination and AD was detected. We provide preliminary indication for a possible association between the ADORA2A rs5760423 genetic polymorphism and AD.

## 1. Introduction

Alzheimer’s disease (AD) is considered to be the most common type of dementia, accounting for over 60% of all dementia diagnoses worldwide [1]. AD is a neurodegenerative disease, with intracellular neurofibrillary tau tangles and β-amyloid (Aβ) extracellular plaques as its neuropathological hallmarks [2]. Several risk factors including genetics are considered to have an impact on developing AD, yet aging is considered to have the strongest impact on the disease [3].

Various studies have been conducted in previous years, exploring a multivariate predicting AD model, which could lead to more precise preventing approaches for AD [4]. However, due to the multifactorial etiology of AD [5], such an effort is far from fully accomplished. In fact, environmental and genetic factors have been reported to influence the manifestations of AD’s signs and symptoms [6].

Heretofore, more than 40 genetic loci have been reported for their impact on AD [7,8]. Although some results from previous genetic studies may be false positives, and the magnitude of associations are not highly precise, this continuous genetic research has expanded the landscape on the pathophysiology of AD [7]. Therefore, ongoing research on AD genetics is of great importance, and, doubtlessly, novel data and hypotheses regarding AD pathogenesis will be evident in the near future.

Adenosine receptor subtype A2a (ADORA2A) is a G-protein-coupled adenosine receptor, with an endogenous ligand that is adenosine [9,10]. ADORA2A is implicated in several pathophysiological processes, including neurogenesis, caffeine metabolism, and synaptic plasticity in glutamatergic synapses [11]. Moreover, the loss of adenosine-receptor-mediated modulation on immune cells has an impact on oxidative-stress-mediated inflammation [12,13]. Cytochrome P450 1A2 (CYP1A2) is an inducible enzyme, and it is considered to be the main caffeine-metabolizing enzyme, while it is implicated in several drugs’ metabolisms as well [14,15]. CYP1A2 is also implicated in several pathophysiological mechanisms of oxidative stress [16,17].

Moderate caffeine intake may have a beneficial effect in a transgenic model of AD-like tau pathology [18]. Moreover, coffee intake may decrease the risk of AD and cognitive decline [19,20]. An elevated serum ADORA2A level has been found in patients with AD, and, aside for that fact, amongst CYP1A2 rs762551 C allele carriers, daily coffee consumption was associated with a decreased risk for dementia [21,22]. While, non-constantly, both the ADORA2A rs5760423 and CYP1A2 rs762551 variants have been associated with PD [23,24,25], CYP1A2 rs762551 polymorphism has also been associated with amyotrophic lateral sclerosis and blepharospasm [26,27]. Despite that, AD and PD are two clinically distinct entities, and there is evidence that they share several pathophysiological, phenotypic, and genetic traits [28] including oxidative stress and neuroinflammation [29,30].

Considering that genetic-based AD studies may provide additional data regarding its pathogenesis; that ADORA2A and CYP1A2 are implicated in pathways such as in oxidative stress and caffeine metabolism, which are associated with AD; and also that the ADORA2A rs5760423 and CYP1A2 rs762551 variants have previously been linked to certain neurodegenerative disorders, which share biochemical and clinical similarities with AD, we performed a case–control study aiming to explore for any potential association between these two genetic variants (ADORA2A rs5760423 and CYP1A2 rs762551) and AD.

## 2. Results

### 2.1. Characteristics of the Included Subjects

We genotyped a cohort of 654 subjects (327 patients with AD and an equal number of healthy controls). The AD group consisted of 33.1% males, with a mean age of blood collection ± standard deviation (SD) = 78.90 ± 8.56 years. The control group consisted of 55.9% males, with a mean age ± SD = 69.71 ± 3.02.

### 2.2. Genotypic Call Rate, Hardy–Weinberg Equilibrium (HWE), and Sample Power

Three and seven samples failed to be genotyped for ADORA2A rs5760423 in healthy controls and AD, respectively. For CYP1A2 rs762551, the respective values were three samples for both health controls and patients with AD. Consequently, the overall genotype call rate was >98.47% (98.47% for ADORA2A rs5760423 and 99.08% for CYP1A2 rs762551). Moreover, we did not detect any deviation from the HWE for either ADORA2A rs5760423 (*p* = 0.82 and *p* = 0.74, for the AD and the healthy control groups, respectively) or CYP1A2 rs762551 (*p* = 0.71 and *p* = 0.63, for the AD and the healthy control groups, respectively). Finally, our sample had 81.1 power to reveal a significant association (*p* < 0.05) between the examined genetic variants and AD, with a minor allele frequency (MAF) allele of 33%, a relative genotype risk of 1.65, and a prevalence of AD equal to 37/100,000, assuming the multiplicative model of inheritance.

### 2.3. Primary Endpoint

#### 2.3.1. Analysis for ADORA2A rs5760423

The MAF (T) was 49% in the AD group and 42% in the healthy control group. The genotypic frequency was 85 (27%), 157 (49%), and 78 (24%) for the G/G, G/T, and T/T in the AD group, respectively. The respective values in the healthy control group were 106 (33%), 161 (50%), and 57 (17%). The total numbers (*n*) and frequencies (%) of the alleles and genotypes of ADORA2A rs5760423 for the patients with AD, the healthy controls, and the whole sample are presented in Table 1.

A statistically significant association was found between ADORA2A rs5760423 and AD. More precisely, the “T” allele was associated with increased AD risk in the recessive (odds ratio (OR) = 1.51 (1.03–2.21)), and log-additive (OR = 1.30 (1.04–1.62)) genetic modes. In the codominant model, the TT genotype was more prevalent compared to the GG genotype (OR = 1.71 (1.09–2.66)), with a marginal *p*-value = 0.06. The statistical significance was maintained after adjustment for sex, and it was also evident in codominant mode (*p* = 0.044). The respective results are presented in Table 2.

#### 2.3.2. Analysis for the CYP1A2 rs762551

The MAF (C) was 33% in the AD group and 37% in the healthy control group. The genotypic frequency was 145 (45%), 141 (44%), and 38 (12%) for the A/A, A/C, and C/C in the AD group, respectively. The respective values in the healthy control group were 131 (40%), 147 (45%), and 46 (14%). The total numbers (*n*) and frequencies (%) of the alleles and genotypes of CYP1A2 rs762551 for the patients with AD, the healthy controls, and the whole sample are presented in Table 3.

No association (*p* > 0.05) was found between CYP1A2 rs762551 and AD in any of the examined genetic models of inheritance. Adjustment for sex could not reveal any statistically significant results (*p* > 0.05). The respective results are presented in Table 4.

### 2.4. Secondary Endpoint (Allele–Allele Combination Analysis)

Four allele–allele combinations were created, (1) rs5760423G-rs762551A (G-A), (2) rs5760423T-rs762551A (T-A), (3) rs5760423G-rs762551C (G-C), and (4) rs5760423T-rs762551C (T-C), with proportions *n* = 444, *n* = 390, *n* = 289, and *n* = 84, respectively. The following comparisons were made, (1) G-A carriers vs. non-G-non-A individuals, (2) T-A carriers vs. non-T-non-A individuals, (3) G-C carriers vs. non-G-non-C individuals, and (4) T-C carriers vs. non-T-non-C individuals. The total numbers of individuals carrying allele–allele combinations of ADORA2A rs5760423 and CYP1A2 rs762551 in healthy controls, in AD cases, and in the whole sample are presented in Table 5.

No association (*p* > 0.05) was found between the any allele–allele combination AD, in either crude or adjusted-for-sex analysis (*p* > 0.05). The respective results are presented in Table 6.

## 3. Discussion

In this study, a novel association between the rs5760423 genetic polymorphism of the ADORA2A gene and the risk for AD is presented. Moreover, CYP1A2 rs762551 was not associated with AD in our study.

The literature data have already revealed that degeneration and synaptic dysfunction is a crucial event, attributable for cognitive impairment, which occurs even before the Aβ plaques and tangle formation; the loss of posterior cingulate gyrus and hippocampus synapses is considered as the main neuropathological altering, especially in AD cases. Consequently, AD could also be characterized as a synaptic-based disease [31]. Indeed, the A2A adenosine receptor has been recognized in contributing to synaptic degeneration, and it was recently associated with AD pathogenesis [32]. Exclusively, an elevated A2A expression has been observed in the hippocampal neurons of AD or aged animal models and also in astrocytes of patients with AD and in aged mice [31]. Additionally, the hyperactivation of A2A receptors results in memory issues and alterations of synaptic biomarkers [31]. It has also been found that overexpression of A2A receptors increases TAU hyperphosphorylation and, subsequently, is related to memory deficits in TAUopathic transgenic mice [33]. Due to the A2A adenosine receptor’s synaptic roles in neuronal injury, neuroinflammation, astrocytes, and microglia, it has been suggested as a potential peripheral biomarker in AD cases and even a possible therapeutic target for these patients [31]. Therefore, it is estimated that our study is in parallel with the existing neuropathologic literature data concerning the ADORA2A gene and AD.

Furthermore, genetic polymorphisms occurring in both the ADORA2A and CYP1A2 genes have already been linked to caffeine-induced impairments in postprandial glycaemia [14]. ADORA2A genetic polymorphisms have also been associated with schizophrenia [34], childhood epilepsy, and predisposition to neurologic comorbidity as well as childhood encephalopathy with febrile status epilepticus [35,36], panic disorder [37], methamphetamine-use disorder susceptibility [38], Gilles de la Tourette syndrome [39], and even habitual caffeine consumption, its derived emotional processing, and the anxiety due to caffeine loss [40,41]. Therefore, one could argue that ADORA2A genetic variants are more likely to be related with the specific signs and symptoms of more than one neurological disorder. Bearing in mind that AD’s underlying mode of inheritance and the multifactorial AD etiology still remain unknown, further studies are of great necessity in order for the risk of AD conferred by ADORA2A rs5760423 to be fully clarified.

Heretofore, several important fundamental research studies from the literature data highlight caffeine’s potential protective effect in AD, yet other evidence, mostly from human studies, reveals no link or even implies that caffeine is a real dementia risk factor [42]. Memory issues are a core manifestation of AD, while neuropsychiatric symptoms can also be manifested,— which can even exist from preclinical early AD stages, though they may be developed in different manners in each case; their common denominator is anxiety, and a study conducted with non-transgenic normal aging mice and familial Alzheimer’s models also showed that long-term usage (even in low doses) of caffeine worsened neophobia, cognitive and emotional flexibility, and anxiety-related behaviors, while providing only a small-scale benefit to memory or learning [43]. On the contrary, another study revealed that caffeine may be associated with a reduced dementia and AD risk, since caffeine can interact with and affect several other mechanisms, including elevated insulin sensitivity and antioxidant capacity [44]. Therefore, one could argue that this heterogeneity in AD studies on caffeine could be explained due to genetic variance.

A wide range of studies have reported that the common genetic polymorphisms of the adenosine receptors have a pivotal role in neurologic and psychiatric diseases as well, while the literature data report some novel associations with neuropsychiatric conditions. Of the human adenosine receptor genes, ADORA2A is a dual-coding gene, and it has the most complex structure, resulting in the largest proteinic molecule, whereas its molecular and bioinformatics analyses showed that the various transcripts encoded the same protein, though displayed tissue-specific expression patterns, and that, generally, the highest expression levels were observed in the basal ganglia, blood, spleen, lung, immune cells, and cerebellum [45]. This means that the SNP studied here is not always expressed at the same way in the specific tissues of all AD patients; however, the association with AD was finally evident. Additionally, ADORA2A-related long noncoding RNAs have been reported to be implicated in several processes, such as neurodegeneration and neurodevelopment, and, thus, to neurological and psychiatric disorders [45].

One of the strengths of the present study is the selection of the genetic polymorphism on biological bases. Apart from this, our study contacted in a clinically well-characterized group of AD patients, with ethnically homogeneity. Nevertheless, no study is completely foolproof; firstly, the already-referred-to ethnically homogeneity of our sample, as the whole sample consisted of white Caucasians, may lead to limited generalizability of our findings in populations with different ancestry, especially given that the association between AD with some genetic variants can only be detected in specific ethnicities. Secondly, the present study’s results have not been adjusted for a potential co-founding of other related genetic risk factors, especially the dopamine receptor D2 (DRD2) rs1110976 genetic variant and APOE4 carriage status. As a matter of fact, a functional relationship among the dopaminergic and the adenosinergic systems has already been discussed; adenosine A2A receptors format heteromeric complexes with DRD2 receptors that can have an impact on cell functions, with activation of A2A decreasing the DRD2 signaling [46]. Moreover, we did not include several known AD risk factors (e.g., years of educations, history of traumatic brain injury, dietary habits, etc.) in our statistical regression models. Consequently, the latent effect of additional AD-related covariates in our results cannot totally be excluded. Furthermore, AD subjects were included without any prior screening for major AD-causative genes [7,8]. Even if the risk for AD is studied here in parallel with these polymorphisms, we do not know if the interaction with low or high caffeine intakes could again alter the risk for the disease; meaning that, we have not assessed the caffeine intakes here.

Future studies should be performed, including with additional data and co-founders, such as age, and other genetic and environmental factors (e.g., the amount of coffee that each participant consumes, body mass index, subjective cognitive decline). Moreover, future studies should also examine the association of ADORA2A rs5760423 with other outcomes in AD patients, such as age at the disease onset, initial phenotypic manifestation, disease progression, and disease severity, so further and more precise correlations can be established. Along with this information, the data for anxiety and other related behavioral manifestations as well as the sleep quality ought to be recorded, along with all the previously discussed potential risk factors that could have an impact on the final results. Finally, epigenetic and other environmental parameters should be examined, as, undeniably, the ADORA2A gene interacts with other molecules, and, finally, they contribute to its own epigenetic mechanisms.

## 4. Materials and Methods

### 4.1. Study Design, Ethics, and Informed Consent

A case–control study was performed aiming to examine any potential association between the ADORA2A rs5760423 and the CYP1A2 rs762551 genetic variants and AD. The research protocol was approved by the local Ethics Committee of the University General Hospital of Larissa (132/17-06-2015). All the included participants (or their close relatives when necessary) granted a written informed consent to participate in the study.

### 4.2. Study Population

We recruited consecutive AD patients admitted to the Neurology Department (outpatient and inpatient clinics) of the General University Hospital of Larissa, a tertiary referral institution located in Central Greece. This current study includes the sample from previously published articles [47,48,49,50,51]. Briefly, all patients diagnosed with probable AD (G30 according to the International Classification of Diseases, 10th Revision) by a senior neurologist, based on the National Institute of Neurological and Communicative Disorders and Stroke/Alzheimer Disease and Related Disorders Association (NINCDS/ADRDA) criteria [52]. Healthy volunteers with free medical history record and normal mini-mental state examination (MMSE) scores (conducted by neuropsychologists) that did not fulfill the criteria for Mild Cognitive Impairment were considered as the healthy control group. These unrelated healthy controls were recruited in General University Hospital of Larissa, mainly from patients’ spouses (not the spouses of the patients with AD), hospital employees, and adult visitors to the hospital.

### 4.3. Laboratory Techniques

We used the salting out method [53], for nuclear DNA to be isolated from peripheral blood leucocytes, from all the included subjects. We genotyped all the samples, for the ADORA2A rs5760423 and CYP1A2 rs762551 genetic variants, using the TaqMan allele specific discrimination assay (Thermo Fisher Scientific, Waltham, MA, USA) on an ABI PRISM 7900 Sequence Detection System, with SDS software (SDS 2.4) (Applied Biosystems, Foster City, CA, USA). A detailed description of the entire method (PCR steps, enzyme activation, denaturation, and annealing/extension) has been previously described [54]. The overall procedure (genotyping and analyses of the results) was performed completely by personnel that were blinded to clinical status of the recruited participants, aiming to mitigate any potential bias.

### 4.4. Quality Assessment

Quality assessment was carried out by setting the threshold of the genotypic call rate (percentage of successfully genotyped samples) at 95%. Moreover, we proceeded to re-genotype a randomly selected 10% of the samples. A 100% concordance with the results from the initial genotyping was revealed. Finally, the chi-squared test was applied to calculate the genotyping results, in order to detect any deviation from HWE [55].

### 4.5. Endpoints

The primary endpoint of the present study was to examine any potential association between the ADORA2A rs5760423 and CYP1A2 rs762551 SOD2 and AD. The secondary endpoint was to examine for any potential effect of the allele–allele combination derived from ADORA2A rs5760423 and CYP1A2 rs762551 on AD.

### 4.6. Statistical Analysis

The main demographic characteristics are expressed as *n* and/or percentages for categorical variables and as means with the respective SD for continuous variables. With the CaTS Power Calculator for Genetic Studies (Center for Statistical Genetics, University of Michigan, Ann Arbor, MI, USA), we calculated the statistical power of the genotyped sample size. The total number (*n*), and the percentages for allelic and genotypic distribution in the whole sample, in AD cases, and in healthy controls were calculated. For the primary endpoint, five genetic modes were assumed (codominant, dominant, recessive, overdominant, and log-additive). The ‘G’ was considered as the reference allele and the ‘T’ as the alternative one for the ADORA2A rs5760423 gene variant. The ‘A’ was considered as the reference allele and the ‘C’ as the alternative one for the CYP1A2 rs762551 gene variant. For the secondary endpoint, allele–allele combinations of both variants were created and examined for their association with AD via logistic regression. The risk for every allele–allele combination was estimated by comparing individuals carrying each combination with those that not carrying it. The effect size for both primary and secondary endpoints was expressed in terms of the ORs with their respective 95% confidence intervals (CIs). An alpha error of 5% (two-tailed *p* < 0.05) was set as the statistical significance threshold. The statistical analysis, for the effect of each polymorphism, was performed with SNPStats software (https://www.snpstats.net/, accessed on 10 February 2022) [55]. The statistical analysis for the allele–allele combination comparisons was performed using IBM SPSS Statistics Software Version 26 (Chicago, IL, USA).

## 5. Conclusions

In conclusion, we provide the preliminary indication for a possible association of the ADORA2A rs5760423 genetic polymorphism with the manifestation of AD. Moreover, this is the first study that reports no association of the CYP1A2 rs762551 genetic variant with the disease. Therefore, this study may be of clinical importance for the diagnostic index of AD, and further, more thorough studies are required for our results to be confirmed again.

## Figures and Tables

**Table 1:** Allelic and genotype frequencies for ADORA2A rs5760423 in healthy controls, in AD cases, and in whole sample.

SNP	Genotypes/ Alleles	Healthy Controls (*n* = 327)	AD (*n* = 327)	Whole Sample (*n* = 654)
rs5760423		*n* (%)	*n* (%)	*n* (%)
Genotype	G/G	106 (33)	85 (27)	191 (30)
	G/T	161 (50)	157 (49)	318 (49)
	T/T	57 (17)	78 (24)	135 (21)
Allele	G	373 (58)	327 (51)	700 (54)
	T	275 (42)	313 (49)	588 (46)

SNP, single nucleotide polymorphism; ADORA2A, adenosine A2a receptor; AD, Alzheimer’s disease.

**Table 2:** Single locus analysis for association between ADORA2A rs5760423 and AD, in codominant, dominant, recessive, overdominant, and log-additive modes.

	Unadjusted Analysis			Adjusted Analysis	
Mode	Genotype	OR (95% CI)	*p*-Value	OR (95% CI)	*p*-Value
Codominant	G/G	1.00	0.06	1.00	0.044
	T/G	1.22 (0.85–1.74)		1.15 (0.79–1.66)	
	T/T	1.71 (1.09–2.66)		1.76 (1.11–2.78)	
Dominant	G/G	1.00	0.087	1.00	0.14
	T/G-T/T	1.34 (0.96–1.89)		1.30 (0.92–1.85)	
Recessive	G/G-T/G	1.00	0.034	1.00	0.017
	T/T	1.51 (1.03–2.21)		1.61 (1.09–2.39)	
Overdominant	G/G-T/T	1.00	0.87	1.00	0.56
	T/G	0.98 (0.72–1.33)		0.91 (0.66–1.25)	
Log-additive	---	1.30 (1.04–1.62)	0.02	1.31 (1.04–1.64)	0.019

ADORA2A, adenosine A2a receptor; AD, Alzheimer’s disease; CI, confidence interval; OR, odds ratio. Adjustment was made for sex as a categorical variable.

**Table 3:** Allelic and genotype frequencies for CYP1A2 rs762551 in healthy controls, in AD cases, and in whole sample.

SNP	Genotypes/ Alleles	Healthy Controls (*n* = 327)	AD (*n* = 327)	Whole Sample (*n* = 654)
rs762551		*n* (%)	*n* (%)	*n* (%)
Genotype	A/A	131 (40)	145 (45)	276 (43)
	A/C	147 (45)	141 (44)	288 (44)
	C/C	46 (14)	38 (12)	84 (13)
Allele	A	409 (63)	431 (67)	840 (65)
	C	239 (37)	217 (33)	456 (35)

SNP, single nucleotide polymorphism; CYP1A2, cytochrome P450 1A2; AD, Alzheimer’s disease.

**Table 4:** Single locus analysis for association between CYP1A2 rs762551 and AD in codominant, dominant, recessive, overdominant, and log-additive modes.

	Unadjusted Analysis			Adjusted Analysis	
Mode	Genotype	OR (95% CI)	*p*-Value	OR (95% CI)	*p*-Value
Codominant	A/A	1.00	0.45	1.00	0.43
	C/A	0.87 (0.62–1.21)		0.90 (0.64–1.26)	
	C/C	0.75 (0.46–1.22)		0.72 (0.44–1.19)	
Dominant	A/A	1.00	0.27	1.00	0.33
	C/A-C/C	0.84 (0.61–1.14)		0.85 (0.62–1.18)	
Recessive	A/A-C/A	1.00	0.35	1.00	0.25
	C/C	0.80 (0.51–1.27)		0.76 (0.47–1.22)	
Overdominant	A/A-C/C	1.00	0.64	1.00	0.85
	C/A	0.93 (0.68–1.26)		0.97 (0.70–1.33)	
Log-additive	---	0.86 (0.69–1.08)	0.21	0.86 (0.68–1.09)	0.21

CYP1A2, cytochrome P450 1A2; AD, Alzheimer’s disease; CI, confidence interval; OR, odds ratio. Adjustment was made for sex as a categorical variable.

**Table 5:** Total numbers of individuals carrying allele–allele combinations of ADORA2A rs5760423 and CYP1A2 rs762551 in healthy controls, in AD cases, and in whole sample.

Combined Allele Carriage		Healthy Controls	AD	Whole Sample
rs5760423	rs762551			
G	A	231	213	444
Non-G	Non-A	11	8	19
T	A	184	206	390
Non-T	Non-A	13	8	21
G	C	158	131	289
Non-G	Non-C	23	32	55
T	C	124	138	262
Non-T	Non-C	38	46	84

ADORA2A, adenosine A2a receptor; CYP1A2, cytochrome P450 1A2; AD, Alzheimer’s disease.

**Table 6:** Analysis for association between the combined allele carriage from ADORA2A rs5760423 and CYP1A2 rs762551 and AD.

	Unadjusted Analysis			Adjusted Analysis	
Combined Allele Carriage					
rs5760423	rs762551	OR (95% CI)	*p*-Value	OR (95% CI)	*p*-Value
G	A	1.27 (0.50–3.21)	0.62 ^1^	1.32 (0.51–3.46)	0.57 ^1^
T	A	1.82 (0.74–4.49)	0.19 ^2^	1.89 (0.75–4.75)	0.18 ^2^
G	C	0.59 (0.33–1.07)	0.082 ^3^	0.57 (0.31–1.03)	0.064 ^3^
T	C	0.91 (0.56–1.51)	0.74 ^4^	0.91 (0.55–1.50)	0.71 ^4^

ADORA2A, adenosine A2a receptor; CYP1A2, cytochrome P450 1A2; AD, Alzheimer’s disease; CI, confidence interval; OR, odds ratio. Adjustment was made for sex as a categorical variable. ^1^ G-A vs. non-G-A; ^2^ T-A vs. non-T-A; ^3^ G-C vs. non-G-C; ^4^ T-C vs. non-T-C.

## Data Availability

The data presented in this study are available on reasonable request from the corresponding author.
